# Knowledge, attitudes, and practices of Chinese anesthesiologists toward difficult airways

**DOI:** 10.1186/s12909-025-07264-x

**Published:** 2025-05-09

**Authors:** Shuang Xie, Wen-Jing Xu, Huan-Liang Wang

**Affiliations:** Department of Anesthesiology, The Second Affiliated Hospital of Hainan Medical University, No.368, Yihai Avenue, Longhua District, Haikou, 570311 Hainan China

**Keywords:** Difficult airway, Anesthesiologists, Knowledge, attitude, practice, Cross-sectional study

## Abstract

**Background:**

Difficult airways (i.e., when a healthcare provider with skills in airway management encounters difficulties when using recognized techniques) require proper training (or supervision), experience, risk assessment, and clinical judgment, but the knowledge, attitude, and practice (KAP) of Chinese anesthesiologists toward difficult airways is poorly known. This study aimed to remedy that problem.

**Methods:**

This cross-sectional study enrolled Chinese anesthesiologists from September 1, 2023, to November 30, 2023. The KAP toward difficult airways was assessed using a questionnaire (Cronbach’s α = 0.705). Scores *≥* 85% indicated good/positive/proactive knowledge/attitudes/practice, 50-75% were moderate, and < 50% were poor. The factors associated with practice were identified by multivariable logistic regression.

**Results:**

A total of 992 questionnaires were included. The participants were 39.89 ± 8.46 years, 52.72% were male, 79.13% were working in tertiary hospitals, and 19.96% had 6–10 years of experience in anesthesia. The mean knowledge, attitude, and practice scores were 18.09 ± 2.46 (69.58% of the maximum), 32.22 ± 2.77 (92.06% of the maximum), and 63.80 ± 5.54 (85.07% of the maximum), respectively. 97.18% of anesthesiologists reported that they frequently assess all patients for the risk of difficult airway and aspiration prior to anesthesia administration or airway management. Notably, however, 30.14% of doctors indicated that they may not confirm the availability of difficult airway equipment in the operating room if the patient has not been evaluated as having a difficult airway. Furthermore, only 41.93% of anesthesiologists reported being able to successfully use awake fiberoptic intubation when managing a difficult airway on a frequent basis. The high attitude scores (Odds Ratio [OR] = 1.234, 95% Confidence Interval [CI]: 1.164–1.307, *P* < 0.001), master’s degree or above (OR = 2.262, 95%CI: 1.539–3.323, *P* < 0.001), and participated in training on difficult airway assessment and management in the past 6 months (OR = 1.943, 95%CI: 1.388–2.720, *P* < 0.001) were more likely to achieve higher practical scores. After adjustment, the SEM showed that knowledge directly influenced attitude (β = 0.338, *P* = 0.011) but not practice (*P* = 0.637); attitude directly influenced practice (β = 0.584, *P* = 0.003).

**Conclusion:**

Anesthesiologists in China have favorable KAP toward difficult airways. Nonetheless, certain practices require enhancement. Since knowledge is associated with attitude and attitude with practice, educational and motivational activities should be designed to improve practice.

**Clinical trial number:**

not applicable.

**Supplementary Information:**

The online version contains supplementary material available at 10.1186/s12909-025-07264-x.

## Background

Airway intubation is a basic technique for critically ill patients requiring mechanical ventilation and patients undergoing general anesthesia for surgery [1]. The difficulty level of a given airway depends upon several factors, including patient characteristics, medical and surgical history, airway examination, clinical context for airway management, and the patient’s status and vital signs [2, 3]. An airway is considered difficult when a healthcare provider with skills in airway management encounters difficulties when using recognized techniques [4, 5]. Healthcare providers should have proper training (or supervision), experience, risk assessment, and clinical judgment when performing airway management [6, 7]. Trained and experienced healthcare providers are expected to succeed in managing basic and straightforward airways without complications. In the real world, airway assessment is highly subjective and will vary among healthcare providers based on training, skills, and experience; furthermore, depending on the patient population, as many as 90% of airways might be unanticipated [8]. Complex airways will require infrequently used techniques and access to teams with special skills and equipment. They will typically require more than one attempt before success, delaying proper patient management in life-threatening situations or resulting in complications [6, 7]. The most common complications are inadvertent esophageal intubation, bronchial intubation, vocal cord displacement, tooth damage, mediastinal intubation, tracheoesophageal fistula, tracheal stenosis, and death, especially for patients requiring emergent ventilation but with intubation failure [6, 7]. Hence, irrespective of the degree of difficulty of an airway, patients requiring airway management will ultimately be successfully managed for proper care [3].

Although many clinicians may have to perform airway management, anesthesiologists perform airway management routinely as part of their practice. In addition, they are the specialists who are called for help with difficult airway management [7, 9, 10]. Recent guidelines provide significant advancements in airway management [11]. Even among anesthesiologists, proper airway management requires knowledge of the techniques and devices available, a positive attitude toward difficult airways, and adequate techniques in given clinical scenarios. Knowledge, attitude, and practice (KAP) studies provide qualitative and quantitative data about the gaps, misconceptions, and misunderstandings that may hinder the optimal performance of a given subject in each population [12, 13]. KAP studies can identify gaps that can be targeted by educational and motivational interventions to improve practice. Results from KAP studies can be used to improve physicians’ practice on a precise subject, which can translate into improved patient outcomes [12–14]. No previous studies performed a comprehensive KAP assessment of anesthesiologists’ KAP toward difficult airways, but some partial data are nonetheless available on specific aspects, mainly practice, from countries such as India, the United Kingdom, and the Republic of Macedonia [15–20]. Therefore, this study aimed to examine the anesthesiologists’ KAP toward difficult airways. Chinese anesthesiologists follow international guidelines on difficult airway management, but access to specific or super-specialized tools can be limited. Hence, assessing the KAP of Chinese anesthesiologists toward airway management is important.

## Methods

### Study design and participants

This cross-sectional study was conducted from September 1, 2023, to November 30, 2023, and enrolled anesthesiologists from multiple hospitals in China. This study was approved by the Institutional Review Board of the Second Affiliated Hospital of Hainan Medical University (LW2023161), and all participants provided written informed consent.

The inclusion criteria were (1) anesthesiologists working in hospitals in China and (2) provided informed consent. Non-practicing physicians during the study period (e.g., sick leave or purely administrative position) were excluded.

### Questionnaire introduction

The questionnaire was designed based on the relevant guidelines and literature [7, 21, 22]. After the initial design, modifications were made based on feedback from four anesthesia experts. The questionnaire underwent two small-scale pilot testing (consisting of 47 and 46 responses, respectively). The final version of the questionnaire **(Appendix)** was obtained following adjustments based on the feedback from pilot testing. The Cronbach’s α coefficient of the pilot test was 0.705, indicating acceptable internal consistency reliability. The participants of the pilot testing were also asked to report any difficulties in understanding the questions to evaluate face value.

The final questionnaire comprised four dimensions: demographic information (education level, gender, job type, institutional nature, professional title, etc.), knowledge dimension, attitude dimension, and practice dimension. The knowledge dimension consisted of 13 questions, with correct answers scoring 2 points, uncertain answers scoring 1 point, and incorrect answers scoring 0 points, resulting in a score range of 0–26 points. The scores for each item were calculated as the total scores of all participants divided by the maximum possible score. The attitude dimension included seven questions, utilizing a 5-point Likert scale ranging from “very positive” (5 points) to “very negative” (1 point), with a score range of 7–35 points. The practice dimension comprised 15 questions, also using a 5-point Likert scale ranging from “always” (5 points) to “never” (1 point), with a score range of 15–75 points. Higher KAP scores indicate better KAP. We used a modified Bloom criterion for categorizing the KAP dimension scores [23]. Scores *≥* 75% were good, 50-75% were moderate, and < 50% were poor. Such cutoffs were used in previous KAP studies [24].

### Questionnaire collection and quality control

Three trained research assistants were involved in this study to apply quality control throughout the entire process. It encompassed the following aspects: comprehensive standard operating procedures that comply with regulations, ethics, and scientific standards, protection of the rights of participants, selection of qualified research centers and researchers, training of research personnel, thorough recording and reporting of experimental data, and collection, storage, and updating of the necessary documents. These measures suggest that the survey adhered to scientific principles was conducted credibly, and maintained high data quality standards.

The hospital selection criteria were not limited by hospital level or province to minimize bias. Forty hospitals were initially considered through convenience sampling (Hainan, Sichuan, Tianjin, Shandong, Guangxi, Guangdong, Henan, Jilin, Zhejiang, Shaanxi, Xinjiang, Jiangsu, and Guizhou, covering all regions of China), but 26 (in nine provinces) finally participated in the study. The proportion of secondary and tertiary questionnaires was determined according to the number of beds. In Chinese hospitals, the number of beds determines the scale and level of the hospital; furthermore, the number of medical staff is assigned according to the number of beds, and the number of anesthesiologists is assigned according to the number of surgeons. Therefore, a hospital with a small number of beds means that there are fewer anesthesiologists, so fewer questionnaires were assigned, and the questionnaires were distributed using the Questionnaire Star platform based on the number of hospital beds (hospital level). Approximately 20 questionnaires were distributed per secondary and lower-level hospitals (including private hospitals), and approximately 60 were distributed per tertiary hospitals.

Informed consent was the first part of the questionnaire and was mandatory to gain access to the questionnaire itself. A given IP address could be used only once to submit a questionnaire. All questions were mandatory for submission. During quality control, questionnaires with excessively short (< 90 s) or long (> 1800 s) response times, numerical errors, logic errors, and filled with all the same answers (e.g., all first choices) were excluded. Those quality control criteria were applied because not carefully reading and answering the questions would affect the quality of the data.

Measures to mitigate the social desirability bias included the use of the Likert scale, cross-questioning of forward and reverse questions, and, before the beginning of the questionnaire, it was made clear to the participants that they needed to accurately feedback their understandings and opinions rather than speculate on the expected answers.

All questionnaires were completed anonymously. Only the IP address was retained to avoid double-completion of questionnaires. All data were accessible only by the investigator and the research team. Once the study was completed, all IP address data were destroyed, and only anonymous data were kept for analysis.

### Statistical analysis

Descriptive analysis was conducted for the demographic data and KAP scores. Continuous variables were described using means ± standard deviations (SDs) and analyzed using the Mann-Whitney U-test (two-level comparisons) or the Kruskal-Wallis H-test (analysis of more than two levels). The Spearman analysis was used to examine the correlation between knowledge, attitudes, and practice scores. Univariable and multivariable logistic regression analyses were performed to explore the factors associated with the KAP dimensions. Structural equation modeling (SEM) was used to analyze the relationships among the dimensions of the questionnaire. The model fit was evaluated using the incremental fit index (IFI), Tucker-Lewis index (TLI), comparative fit index (CFI), root mean square error of approximation (RMSEA), and chi-square minimum divided by the degrees of freedom (CMIN/DF). The mediating role of attitudes in knowledge and practice was assessed using Bootstrap analysis. Statistical analyses were conducted using SPSS 26.0 (IBM, Armonk, NY, USA). Two-sided P-values < 0.05 were considered statistically significant.

## Results

### Characteristics of the participants

A total of 1050 questionnaires were distributed, and 1021 questionnaires were returned. Of the 1021 questionnaires, four were excluded for taking < 90 s to complete, 23 for > 1800 s to complete, one with age reported as 137 years, and one with reported work experience > 100 years. Hence, 992 valid questionnaires were included. Cronbach’s α was 0.700 for the entire study population, and the Kaiser-Meyer-Olkin (KMO) was 0.839. The participants were 39.89 ± 8.46 years. For each characteristic, the highest frequency was observed for males (52.72%), master’s degree and above (50.20%), intermediate title (42.84%), tertiary hospitals (79.13%), with 6–10 years of experience in anesthesia (19.96%), did not participate in difficult airway training in the past 6 months (56.65%), did not encounter patient death caused by difficult airway (87.90%), encountered cases where surgery was stopped due to difficult airways (61.09%), encountered cases of difficult airways and successfully rescued (90.83%), rarely encounter difficult airways (60.69%), no death related to difficult airways in the department in the past 6 months (96.77%), and no cases where the surgery was stopped in the past 6 months in the department (80.14%) (Table 1).

**Table 1:** Characteristics of the participants and KAP scores

	*n* (%)	Knowledge	*P*	Attitude	*P*	Practice	*P*
*n* = 992							
Total score		18.09 ± 2.46		32.22 ± 2.77		63.80 ± 5.54	
Age	39.89 ± 8.46						
Gender			0.001		< 0.001		0.252
Male	523 (52.72)	17.85 ± 2.50		31.90 ± 2.95		63.89 ± 5.59	
Female	469 (47.28)	18.36 ± 2.37		32.59 ± 2.50		63.69 ± 5.49	
Education			0.094		0.001		0.004
Bachelor’s/associate degree	494 (49.80)	17.93 ± 2.51		31.92 ± 2.97		63.16 ± 6.02	
Master’s and above	498 (50.20)	18.25 ± 2.39		32.53 ± 2.52		64.43 ± 4.94	
Title			< 0.001		0.712		< 0.001
Junior and below	196 (19.76)	17.49 ± 2.70		31.94 ± 3.13		62.22 ± 6.39	
Intermediate	425 (42.84)	18.09 ± 2.47		32.25 ± 2.74		63.70 ± 5.33	
Associate senior and above	371 (37.40)	18.40 ± 2.24		32.35 ± 2.59		64.74 ± 5.08	
Nature of institution			0.184		< 0.001		0.271
Public Level Two and below	173 (17.44)	17.78 ± 2.63		31.58 ± 2.97		62.82 ± 6.58	
Public Level Three	785 (79.13)	18.17 ± 2.43		32.38 ± 2.72		64.01 ± 5.29	
Private Hospital	34 (3.43)	17.74 ± 2.06		31.82 ± 2.38		63.82 ± 4.90	
Years of experience in anesthesia			0.179		0.448		< 0.001
0–5	194 (19.56)	17.76 ± 2.52		32.25 ± 2.97		62.36 ± 5.88	
6–10	198 (19.96)	17.90 ± 2.58		32.03 ± 2.95		63.01 ± 5.81	
11–15	173 (17.44)	18.10 ± 2.45		32.22 ± 2.90		63.73 ± 5.62	
16–20	164 (16.53)	18.28 ± 2.49		32.35 ± 2.47		64.96 ± 5.06	
21–25	102 (10.28)	18.39 ± 2.23		32.68 ± 2.39		64.49 ± 5.31	
25–30	105 (10.58)	18.43 ± 2.34		31.98 ± 2.84		64.85 ± 4.96	
More than 30	56 (5.65)	18.11 ± 2.18		32.09 ± 2.25		65.11 ± 4.39	
Participated in training on difficult airway assessment and management in the past 6 months			0.417		0.028		< 0.001
Yes	430 (43.35)	18.01 ± 2.50		32.39 ± 2.81		64.90 ± 5.21	
No	562 (56.65)	18.15 ± 2.42		32.10 ± 2.73		62.95 ± 5.63	
Encountered cases where patient death was caused by a difficult airway			0.792		0.190		0.312
Yes	120 (12.10)	17.99 ± 2.59		31.87 ± 2.97		64.13 ± 5.64	
No	872 (87.90)	18.10 ± 2.44		32.27 ± 2.74		63.75 ± 5.53	
Encountered cases where surgery was stopped due to a difficult airway			0.341		0.140		0.002
Yes	606 (61.09)	18.15 ± 2.41		32.35 ± 2.64		64.28 ± 5.14	
No	386 (38.91)	17.99 ± 2.53		32.03 ± 2.95		63.03 ± 6.04	
Encountered cases of difficult airways and successfully rescued			0.078		< 0.001		< 0.001
Yes	901 (90.83)	18.14 ± 2.41		32.35 ± 2.67		64.10 ± 5.21	
No	91 (9.17)	17.59 ± 2.85		31.02 ± 3.38		60.82 ± 7.54	
Frequency of encountering difficult airway patients in your department			0.188		0.069		0.324
Rarely	602 (60.69)	18.01 ± 2.42		32.13 ± 2.72		63.68 ± 5.78	
Sometimes	311 (31.35)	18.29 ± 2.46		32.33 ± 2.82		63.76 ± 5.24	
Often	79 (7.96)	17.91 ± 2.69		32.56 ± 2.91		64.82 ± 4.65	
Death due to a difficult airway in the past 6 months in the department			0.031		0.399		0.477
Yes	32 (3.23)	17.09 ± 3.19		31.00 ± 4.38		63.78 ± 7.51	
No	960 (96.77)	18.12 ± 2.42		32.27 ± 2.69		63.80 ± 5.47	
Cases where surgery was stopped due to a difficult airway in the past 6 months in the department			0.646		0.129		0.156
Yes	197 (19.86)	18.15 ± 2.73		32.32 ± 3.00		64.20 ± 5.51	
No	795 (80.14)	18.08 ± 2.38		32.20 ± 2.71		63.70 ± 5.54	

### Knowledge

The mean knowledge score was 18.09 ± 2.46 (on a maximum of 26, 69.58%). For knowledge, gender showed a marked difference (*P* = 0.001), with females scoring higher (18.36 ± 2.37) than males (17.85 ± 2.50). Title (*P* < 0.001) and participation in difficult airway training (*P* = 0.078) also influenced knowledge, though training did not reach significance., (Table 1). The highest knowledge accuracy was observed for K6 (97.58%; “Informing patients or their families in advance of the risks and procedures of difficult airway management is part of preparing for difficult airways.”), while the lowest knowledge accuracy was observed for K1 (3.02%; “Airway risk assessment and airway examination before anesthesia or airway management are mainly based on physical examination and additional special assessment methods.”) (**Table ****S1****)**.

### Attitudes

The mean attitude score was 32.22 ± 2.77 (on a maximum of 35, 92.06%). Attitude scores differed significantly by gender (*P* < 0.001), education (*P* = 0.001), institution type (*P* < 0.001), and successful rescue of difficult airway cases (*P* < 0.001). Notably, higher education levels (Master’s/above: 32.53 ± 2.52 vs. Bachelor’s/associate: 31.92 ± 2.97) and public Level Three institutions (32.38 ± 2.72 vs. Level Two/below: 31.58 ± 2.97) were associated with better attitudes. (Table 1). The highest attitude score was observed for A1 (98.79% - comprising 90.22% strongly agreeing and 8.57% agreeing; “You believe that assessing the risk of difficult airway and aspiration before anesthesia or airway management is crucial.”) while the lowest attitude score was observed for A3 (77.62% t—39.01% strongly disagreeing and 38.61%; “You strongly resist participating in the anesthesia and management of difficult airway patients due to concerns about causing injury from improper handling. (negative)”) (**Table ****S2****)**.

### Practices

The mean practice score was 63.80 ± 5.54 (on a maximum of 75, 85.07%). For practice, significant differences emerged by title (*P* < 0.001), years of anesthesia experience (*P* < 0.001), recent training (*P* < 0.001), encountering surgery-stopped cases (*P* = 0.002), and successful rescue experiences (*P* < 0.001). Participants with associate senior titles (64.74 ± 5.08) and those with > 30 years of experience (65.11 ± 4.39) scored highest in practice, while training attendees (64.90 ± 5.21) outperformed non-attendees (62.95 ± 5.63). No significant differences were observed in practice scores by gender, institution type, or most clinical encounter frequencies. (Table 1). The highest practice score was observed for P1 (97.18% - comprising 77.32% always and 19.86% often; “Before administering anesthesia or airway management, you assess the risk of difficult airway and aspiration for all patients.”), while the lowest practice score was observed for P12 (41.93% - comprising 13% always and 29.13% often; “When handling difficult airways, you successfully use awake fiberoptic intubation. (positive)”). Of note, for P7, only 48.69% (12.3% always and 17.84% often) were proactive regarding “If a patient is not assessed as having a difficult airway, you may not confirm whether the operating room is equipped with difficult airway equipment before administering anesthesia” (**Table ****S3****)**.

### Correlations

The knowledge scores were correlated to the attitude scores (*r* = 0.177, *P* < 0.001) but not to the practice scores (*r* = 0.039, *P* = 0.216). The attitude scores were correlated to the practice scores (*r* = 0.282, *P* < 0.001) (Table 2).

**Table 2:** Correlation analysis of KAP scores

	Knowledge	Attitude	Practice
Knowledge	1		
Attitude	0.177 (*P* < 0.001)	1	
Practice	0.039 (*P* < 0.216)	0.282 (*P* < 0.001)	1

### Multivariable analysis for practice

The attitude scores (OR = 1.234, 95%CI: 1.164–1.307, *P* < 0.001), master’s degree or above (OR = 2.262, 95%CI: 1.539–3.323, *P* < 0.001), and participated in training on difficult airway assessment and management in the past 6 months (OR = 1.943, 95%CI: 1.388–2.720, *P* < 0.001) were independently associated with the practice scores (Table 3).

**Table 3:** Factors of practice based univariable and multivariable logistic regression

	Univariable logistic regression		Multivariable logistic regression	
	OR (95%CI)	*P*	OR (95%CI)	*P*
Knowledge score	1.065 (1.004–1.130)	0.036	0.977 (0.914–1.045)	0.500
Attitude score	1.235 (1.172–1.301)	< 0.001	1.234 (1.164–1.307)	< 0.001
Age	1.039 (1.020–1.058)	< 0.001	1.053 (0.996–1.113)	0.067
Gender				
Male	1.181 (0.884–1.577)	0.259		
Female	ref			
Education				
Bachelor’s /associate degree	ref		ref	
Master’s and above	1.917 (1.426–2.577)	< 0.001	2.262 (1.539–3.323)	< 0.001
Title				
Junior and below	ref		ref	
Intermediate	1.792 (1.244–2.582)	0.002	1.192 (0.669–2.123)	0.550
Associate senior and above	2.587 (1.749–3.828)	< 0.001	1.194 (0.553–2.580)	0.652
Nature of institution				
Public Level Two and below	ref		ref	
Public Level Three	1.514 (1.054–2.175)	0.025	0.980 (0.622–1.544)	0.932
Private Hospital	1.261 (0.551–2.882)	0.583	1.231 (0.510–2.968)	0.644
Years of experience in anesthesia				
0–5	ref		ref	
6–10	1.188 (0.775–1.823)	0.430	1.160 (0.607–2.218)	0.653
11–15	1.536 (0.971–2.429)	0.067	1.173 (0.501–2.742)	0.713
16–20	2.498 (1.500-4.159)	< 0.001	1.997 (0.700-5.703)	0.196
21–25	1.790 (1.024–3.130)	0.041	0.973 (0.279–3.401)	0.966
25–30	2.092 (1.181–3.706)	0.011	1.148 (0.273–4.825)	0.851
More than 30	2.014 (0.976–4.154)	0.058	0.942 (0.175–5.075)	0.945
Participated in training on difficult airway assessment and management in the past 6 months				
Yes	1.847 (1.362–2.503)	< 0.001	1.943 (1.388–2.720)	< 0.001
No	ref		ref	
Encountered cases where patient death was caused by a difficult airway				
Yes	1.141 (0.724–1.799)	0.570		
No	ref			
Encountered cases where surgery was stopped due to a difficult airway				
Yes	1.373 (1.024–1.840)	0.034	1.026 (0.720–1.462)	0.888
No	ref		ref	
Encountered cases of difficult airways and successfully rescued				
Yes	2.069 (1.320–3.245)	0.002	0.996 (0.582–1.706)	0.990
No	ref		ref	
Frequency of encountering difficult airway patients in your department				
Rarely	ref		ref	
Sometimes	1.234 (0.896-1.700)	0.198	1.129 (0.779–1.638)	0.521
Often	2.038 (1.074–3.867)	0.029	1.682 (0.826–3.426)	0.152
Cases of death due to difficult airway in the past 6 months in the department				
Yes	0.978 (0.433–2.206)	0.957		
No	ref			
Cases where surgery was stopped due to a difficult airway in the past 6 months in the department				
Yes	1.522 (1.030–2.251)	0.035	1.545 (0.984–2.424)	0.059
No	ref		ref	

### Structural equation modeling

According to structural equation model (SEM) analysis (Figure S1), the model goodness of fit was significantly improved in the baseline adjusted model (Figure S1C), and indicators such as RMSEA (0.056) and CFI (0.728) reached acceptable levels (Table S4). The results showed that knowledge had a significant direct positive effect on attitude (β = 0.338, *p* = 0.011; Table 4), while the direct effect of knowledge on practice was not significant (β = 0.041, *p* = 0.637). As a key mediating variable, attitude showed a strong direct effect on practice (β = 0.584, *p* = 0.003), and mediated the indirect effect of knowledge on practice through this path (indirect effect β = 0.197, *p* = 0.006; 95%CI:0.119–0.303), indicating that attitudes play a central role in the translation of knowledge into practice behavior (Table 4). In addition, experience in anesthesia (β = 0.026, *p* < 0.001), participation in training (β = 0.089, *p* < 0.001) and successful handling of difficult airway cases (β = 0.092, *p* = 0.006) directly improved practice level, and the latter significantly enhanced attitude (β = 0.074, *p* = 0.007; Table S5). In the measurement model, all observed variables of attitudes (A1-A7) and practices (P1-P15) were significantly correlated with their corresponding latent variables (*p* < 0.001), verifying the validity of the scale structure (Table S5).

**Table 4:** Bootstrap analysis of mediating effect significance test for the final model

Model paths (model graph after MI index adjustment)	Standardized direct effects	*P*	95%CI	Standardized indirect effects	*P*	95%CI
Knowledge → Attitude	0.351	0.014	0.206–0.491			
Knowledge → Practice	0.044	0.622	-0.125-0.173			
Attitude → Practice	0.604	0.005	0.516–0.726			
Knowledge → Practice				0.212	0.006	0.133–0.324
**Model paths (model graph after baseline adjustment added)**	**Standardized direct effects**	**P**	**95%CI**	**Standardized indirect effects**	**P**	**95%CI**
Knowledge → Attitude	0.338	0.011	0.206–0.477			
Knowledge → Practice	0.041	0.637	-0.129-0.157			
Attitude → Practice	0.584	0.003	0.497–0.717			
Knowledge → Practice				0.197	0.006	0.119–0.303

## Discussion

Anesthesiologists performing airway management should have proper training (or supervision), experience, risk assessment, and clinical judgment when encountering difficult airways. Improper management of difficult airways can have dire consequences for the patients. No studies comprehensively examined the KAP of anesthesiologists toward difficult airways. Therefore, this study aimed to investigate the anesthesiologists’ KAP toward difficult airways. The study showed that anesthesiologists in China have favorable KAP toward difficult airways. Some knowledge and attitude items were identified as suboptimal. Since knowledge is associated with attitude and attitude with practice, educational and motivational activities should be designed to improve practice and maybe patient outcomes. Education should focus on the definition and assessment of difficult airways, the Mallampati score, and the risk of excessive attempts. The practice of fiberoptic intervention should also be improved. Such interventions could include lectures, podcasts, interactive websites, and practical training. The KAP of the participants could be evaluated before and after the intervention. The impact of the intervention on the patient outcomes could be evaluated, but it can be hypothesized that such interventions could translate into a higher success rate of intubation and/or lower complication rates.

Establishing and maintaining proper airway management is critical in many patients but may be challenging and can lead to a catastrophe in case of failure [7, 22]. Previous studies examined some aspects of the KAP toward difficult airways but not comprehensively. Indeed, in India, Rajesh et al. [15] reported that anesthesiologists routinely performed preoperative assessments, and they thought that hospitals should keep an inventory of various tools for intubation. As assessed in P7, the present study showed that only about half of the participants thought the operating rooms should have all the equipment and tools for difficult airways. Still, the question was about the operating room, not the hospital itself, but the time needed to bring special equipment from storage to the operating room can be long enough to cost lives. In Denmark, Kristensen et al. [16] observed that anesthesiologists have room for improved knowledge and skill of difficult airways. In the present study of Chinese anesthesiologists, knowledge was moderate, but attitudes were positive, and practice was proactive. The present study highlights the necessity for enhancing knowledge regarding methods for preoperative airway assessment, the Mullampati score, use of video laryngoscopy, and adherence to guideline recommendations regarding the number of intubation attempts. Of note, only 42% of the participants reported using fiberoptic intubation, a recognized technique for difficult airway management [25, 26]. Why the anesthesiologists in China do not use fiberoptic intubation remains to be explored, but a study from Canada also showed a declining use of fiberoptic intubation over a 6-year period [27]. The increasing role of video laryngoscopy, declining education and training, elective use on patients, and/or cost may contribute to the lack of use of fiberoptic intubation in China. Furthermore, this study revealed that 20% of anesthesiologists were strongly resisting participating in difficult airway management, which is a worrying problem. However, the reason for their resistance is that they are not confident in their own skills and worry about the damage caused by improper handling of patients with difficult airways. Therefore, we suggest that these doctors should strengthen their skills training and seek timely help from colleagues when encountering emergency airway management. It will be explored in the future.

A study from Brazil showed that residents’ knowledge about difficult airways increased with residency time [17]. Kristensen et al. [16] also advocate that anesthesiologists be trained on the management algorithms and special intubation techniques. Still, experience is required since a study showed that residents gained most of their experience when managing emergent intubations outside of the operating room instead of simulators, with a sharper increase in knowledge and algorithm adherence [18]. On the other hand, a study on German healthcare providers showed that a simulator-based training intervention could effectively improve the participants’ attitudes and behavior toward difficult airways [20]. A study in Macedonia showed that airway assessment, adequate training, experience, and availability of essential equipment were critical to managing difficult airways [19]. Nevertheless, the SEM and multivariable analyses showed that knowledge was associated with attitude, that attitude was associated with practice, and that knowledge was associated with practice through attitude. Although knowledge had no direct influence on practice, it had an indirect influence through attitudes. Hence, improving knowledge and attitude should also improve the practice. Although the study showed a statistically significant association between knowledge and attitude and behavior change, the strength of the association was only below moderate. This means that improving knowledge and positive attitudes alone are not enough to fully translate into actual behavior change. Therefore, in addition to imbuing knowledge and fostering positive attitudes, other key elements such as providing practice opportunities, enhancing self-efficacy, and creating a supportive environment need to be focused on when designing an intervention or educational programme. A combination of these measures can be more effective in driving desired behavior change.

Furthermore, higher education levels correlated with improved practice scores, likely attributed to more extensive training. Additionally, engagement in difficult airway training within the past 6 months was associated with enhanced practice, possibly due to the refresher effect and revisiting current guidelines. Both undergraduate and postgraduate students are engaged in the same type of work in clinical practice. Still, as 79% of the questionnaire came from tertiary hospitals, the proportion of postgraduate students was relatively high.

Although the hospital levels were not independently associated with practice, they have different infrastructures or policies that could influence training and experience. Indeed, primary hospitals are community hospitals that perform the routine care of patients and the management of common chronic conditions. Secondary hospitals are regional hospitals that can handle more complicated conditions and common surgeries. Tertiary hospitals are specialized academic hospitals that handle complex conditions and surgeries. Of course, novel technologies are more likely to be experimented first in tertiary hospitals than in primary hospitals. Nevertheless, access to novel technologies is dependent upon the costs and the will of the stakeholders, which could explain the lower use of fiberoptic intubation.

A strength of this study is the inclusion of multiple hospitals across the country. Still, the study also had limitations. Only 26 hospitals were selected, representing a very small proportion of the hospitals in China, resulting in fewer participants when considering the number of anesthesiologists in China. On the other hand, the sample size requirement of 5–10 participants times the number of items (*n* = 35) [28], i.e., 175–350 participants, was satisfied. The study used convenience sampling, which can introduce a selection bias, but the general distribution of the participants across the hospital levels was representative of the actual distribution of the anesthesiologists in China. Nevertheless, rural area representativeness could be insufficient. The study was cross-sectional, and the results represent KAP at a single time point. Nevertheless, the results could serve as a historical baseline for future intervention studies. In addition, cross-sectional designs do not allow the analysis of causality. A SEM analysis was performed to downplay the issue. In a SEM analysis, “directional associations” can be inferred based on predefined hypotheses, but the results have to be interpreted with caution because such directional associations, which are not causality, are statistically inferred and based on hypotheses defined arbitrarily [29–31]. All KAP studies are at risk of the social desirability bias, which entails that the participants can be tempted to answer what they know they should think and do instead of what they are thinking and doing [32, 33]. Considering that the knowledge scores were relatively high, that bias is possible. Although IFI, TLI, and CFI are slightly below the criterion of 0.8, our model performs well on CMIN/DF and RMSEA with 4.053 and 0.056, respectively, indicating a high model fit overall. We acknowledge the importance of IFI, TLI, and CFI indicators and agree that model improvement remains necessary. Future studies will increase the sample size, which could allow the addition of more paths, such as hospital policy and hospital support.

## Conclusion

In conclusion, anesthesiologists in China have favorable KAP toward difficult airways. Some knowledge and attitude items were identified as suboptimal. Since knowledge is associated with attitude and attitude with practice, educational and motivational activities should be designed to improve practice and maybe patient outcomes. Future studies could examine the impact of such interventions and correlate the KAP with patient outcomes. Policymakers should include difficult airway training in the continuing education and training curricula.

## Electronic supplementary material

Below is the link to the electronic supplementary material.

Supplementary Material 1

Supplementary Material 2

Supplementary Material 3

Supplementary Material 4

Supplementary Material 5

Supplementary Material 6

Supplementary Material 7

## Data Availability

All data generated or analyzed during this study are included in this article and supplementary information files.

## Abbreviations

KAP: Knowledge, attitude, and practice
SDs: Standard deviations
SEM: Structural equation modeling
KMO: Kaiser-Meyer-Olkin

## References

[1] Avva U, Lata JM, Kiel J. Airway management. StatPearls. Treasure Island (FL) ineligible companies. Disclosure: Julie Lata declares no relevant financial relationships with ineligible companies. Disclosure: John Kiel declares no relevant financial relationships with ineligible companies.2024.

[2] Huitink JM, Bouwman RA. The myth of the difficult airway: airway management revisited. Anaesthesia. 2015;70(3):244–9. 25511477 10.1111/anae.12989

[3] Jung H. A comprehensive review of difficult airway management strategies for patient safety. Anesth Pain Med (Seoul). 2023;18(4):331–9. 37919917 10.17085/apm.23123 PMC10635845

[4] Apfelbaum JL, Hagberg CA, Caplan RA, Blitt CD, Connis RT, Nickinovich DG, et al. Practice guidelines for management of the difficult airway: an updated report by the American society of anesthesiologists task force on management of the difficult airway. Anesthesiology. 2013;118(2):251–70. 23364566 10.1097/ALN.0b013e31827773b2

[5] Law JA, Broemling N, Cooper RM, Drolet P, Duggan LV, Griesdale DE, et al. The difficult airway with recommendations for management–part 1–difficult tracheal intubation encountered in an unconscious/induced patient. Can J Anaesth. 2013;60(11):1089–118. 24132407 10.1007/s12630-013-0019-3 PMC3825644

[6] Dorsett M, Panchal AR, Stephens C, Farcas A, Leggio W, Galton C, et al. Prehospital airway management training and education: an NAEMSP position statement and resource document. Prehosp Emerg Care. 2022;26(sup1):3–13. 35001822 10.1080/10903127.2021.1977877

[7] Apfelbaum JL, Hagberg CA, Connis RT, Abdelmalak BB, Agarkar M, Dutton RP, et al. 2022 American society of anesthesiologists practice guidelines for management of the difficult airway. Anesthesiology. 2022;136(1):31–81. 34762729 10.1097/ALN.0000000000004002

[8] Norskov AK, Rosenstock CV, Wetterslev J, Astrup G, Afshari A, Lundstrom LH. Diagnostic accuracy of anaesthesiologists’ prediction of difficult airway management in daily clinical practice: a cohort study of 188 064 patients registered in the Danish anaesthesia database. Anaesthesia. 2015;70(3):272–81. 25511370 10.1111/anae.12955

[9] Grissom TE, Samet RE. The anesthesiologist’s role in teaching airway management to nonanesthesiologists: who, where, and how. Adv Anesth. 2020;38:131–56. 34106831 10.1016/j.aan.2020.08.002 PMC7534755

[10] Collins S, Coughlin M, Daniero J. Who should manage a patient’s airway?? AMA J Ethics. 2020;22(4):E276–82. 32345419 10.1001/amajethics.2020.276

[11] Gomez-Rios MA, Sastre J, Mayo-Yanez M, Serrano-Moraza A. A new perspective on managing the difficult airway: the guidelines of the Spanish societies of anesthesiology, reanimation and pain therapy (SEDAR), emergency medicine (SEMES) and otorhinolaryngology and head and neck surgery (SEORL-CCC). Emergencias. 2024;36(4):303–8. 39234836 10.55633/s3me/039.2024

[12] Andrade C, Menon V, Ameen S, Kumar Praharaj S. Designing and conducting knowledge, attitude, and practice surveys in psychiatry: practical guidance. Indian J Psychol Med. 2020;42(5):478–81. 33414597 10.1177/0253717620946111 PMC7750837

[13] World Health Organization. Advocacy, communication and social mobilization for TB control: a guide to developing knowledge, attitude and practice surveys. http://whqlibdoc.who.int/publications/2008/9789241596176_eng.pdf. Accessed November 22, 20222008.

[14] Kaliyaperumal K. Guideline for conducting a knowledge, attitude and practice AECS Illumination. 2004;4:7–9.

[15] Rajesh MC, Suvarna K, Indu S, Mohammed T, Krishnadas A, Pavithran P. Current practice of difficult airway management: A survey. Indian J Anaesth. 2015;59(12):801–6. 26903674 10.4103/0019-5049.171571 PMC4743304

[16] Kristensen MS, Moller J. Airway management behaviour, experience and knowledge among Danish anaesthesiologists–room for improvement. Acta Anaesthesiol Scand. 2001;45(9):1181–5. 11683672 10.1034/j.1399-6576.2001.450921.x

[17] Imbelloni LE, Paes Ferreira BT, Alabduljabbar N, Viana EP, Sakamoto JW, Austo de Araujo A, et al. The level of knowledge of anesthesiologist residents about difficult airway H management. Intl J Med Res Health Sci. 2021;10(S2):1–7.

[18] Rochlen LR, Housey M, Gannon I, Mitchell S, Rooney DM, Tait AR, et al. Assessing anesthesiology residents’ out-of-the-operating-room (OOOR) emergent airway management. BMC Anesthesiol. 2017;17(1):96. 28709415 10.1186/s12871-017-0387-2 PMC5512836

[19] Kuzmanovska B, Shosholcheva M, Kartalov A, Jovanovski-Srceva M, Gavrilovska-Brzanov A. Survey of current difficult airway management practice. Open Access Maced J Med Sci. 2019;7(17):2775–9. 31844435 10.3889/oamjms.2019.673 PMC6901844

[20] Russo SG, Eich C, Barwing J, Nickel EA, Braun U, Graf BM, et al. Self-reported changes in attitude and behavior after attending a simulation-aided airway management course. J Clin Anesth. 2007;19(7):517–22. 18063206 10.1016/j.jclinane.2007.04.007

[21] Law JA, Duggan LV, Asselin M, Baker P, Crosby E, Downey A, et al. Canadian airway focus group updated consensus-based recommendations for management of the difficult airway: part 2. Planning and implementing safe management of the patient with an anticipated difficult airway. Can J Anaesth. 2021;68(9):1405–36. 34105065 10.1007/s12630-021-02008-z PMC8186352

[22] Frerk C, Mitchell VS, McNarry AF, Mendonca C, Bhagrath R, Patel A, et al. Difficult airway society 2015 guidelines for management of unanticipated difficult intubation in adults. Br J Anaesth. 2015;115(6):827–48. 26556848 10.1093/bja/aev371 PMC4650961

[23] Bloom BS. Learning for Mastery. Instruction and Curriculum. Regional Education Laboratory for the Carolinas and Virginia, Topical Papers and Reprints, Number 1. Evaluation Comment. 1968;1(2):n2.

[24] Ahlgren M, Funk T, Marimo C, Ndiaye C, Alfvén T. Management of Noma: practice competence and knowledge among healthcare workers in a rural district of Zambia. Global Health Action. 2017;10(1):1340253. 28678680 10.1080/16549716.2017.1340253 PMC5533138

[25] Wong J, Lee JSE, Wong TGL, Iqbal R, Wong P. Fibreoptic intubation in airway management: a review Article. Singap Med J. 2019;60(3):110–8. 10.11622/smedj.2018081 PMC6441687 30009320

[26] Ahmad I, El-Boghdadly K, Bhagrath R, Hodzovic I, McNarry AF, Mir F, et al. Difficult airway society guidelines for awake tracheal intubation (ATI) in adults. Anaesthesia. 2020;75(4):509–28. 31729018 10.1111/anae.14904 PMC7078877

[27] Law JA, Thana A, Milne AD. The incidence of awake tracheal intubation in anesthetic practice is decreasing: a historical cohort study of the years 2014–2020 at a single tertiary care institution. Can J Anaesth. 2023;70(1):69–78. 36289151 10.1007/s12630-022-02344-8 PMC9607858

[28] Ni P, Chen JL, Liu N. Sample size Estimation for quantitative studies in nursing research. 2010;45(04):378–80.

[29] Beran TN, Violato C. Structural equation modeling in medical research: a primer. BMC Res Notes. 2010;3:267. 20969789 10.1186/1756-0500-3-267 PMC2987867

[30] Fan Y, Chen J, Shirkey G. Applications of structural equation modeling (SEM) in ecological studies: an updated review. Ecol Process. 2016;5:19.

[31] Kline RB. Principles and practice of structural equation modeling (Fifth Edition). New York: The Guilford Press; 2023.

[32] Bergen N, Labonte R. Everything is perfect, and we have no problems: detecting and limiting social desirability Bias in qualitative research. Qual Health Res. 2020;30(5):783–92. 31830860 10.1177/1049732319889354

[33] Latkin CA, Edwards C, Davey-Rothwell MA, Tobin KE. The relationship between social desirability bias and self-reports of health, substance use, and social network factors among urban substance users in Baltimore, Maryland. Addict Behav. 2017;73:133–6. 28511097 10.1016/j.addbeh.2017.05.005 PMC5519338

