# Supplementary figures and images for: Knowledge, attitudes, and practices of Chinese anesthesiologists toward difficult airways
Source: BMC Med Educ. 2025 May 9;25:683. doi: 10.1186/s12909-025-07264-x (PMC12065192; doi:10.1186/s12909-025-07264-x)

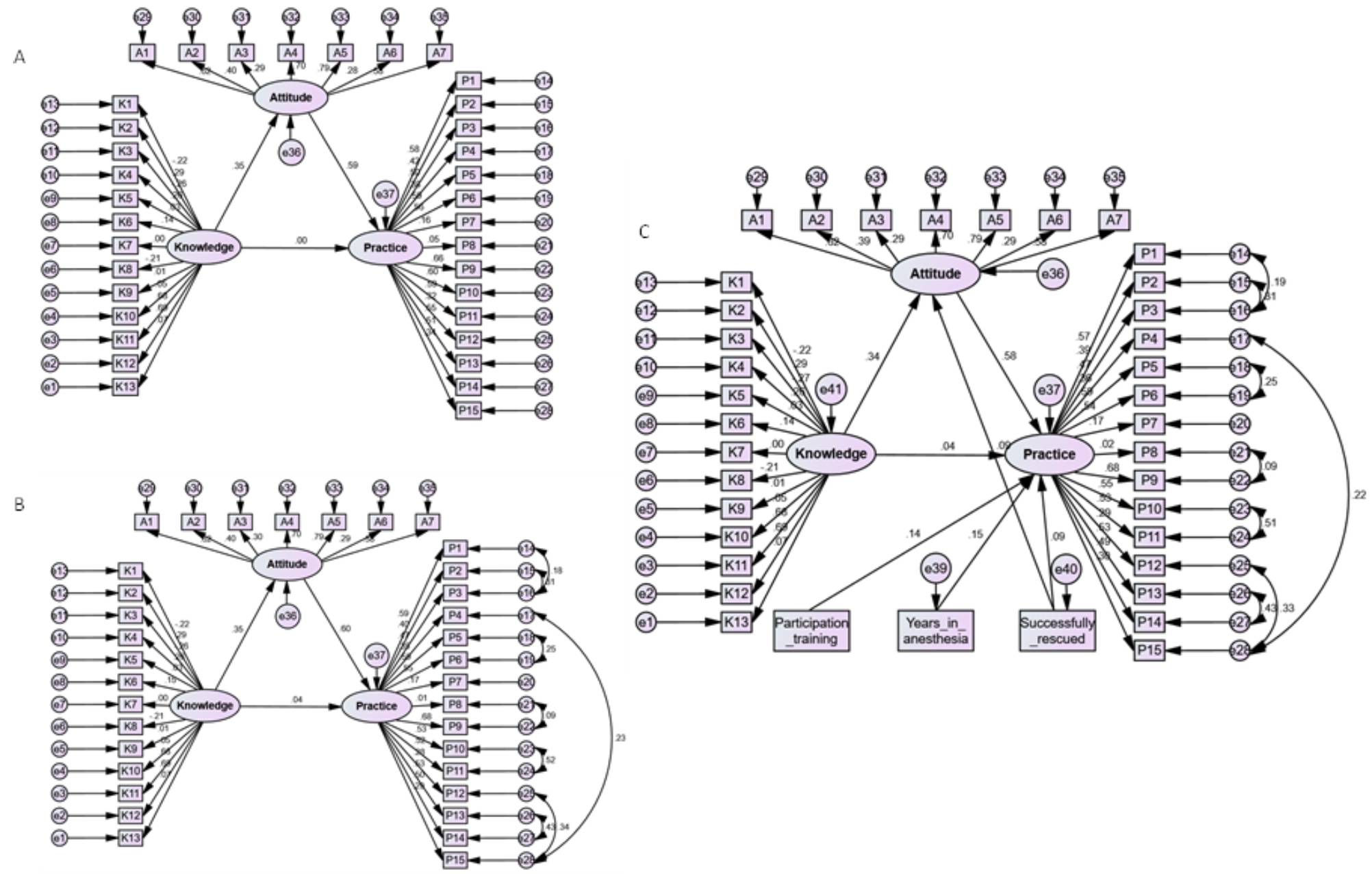

Supplement: Supplementary file 1 — Supplementary Material 1 [file 12909_2025_7264_MOESM1_ESM.jpg]
