# Supplementary material for: Knowledge, attitudes, and practices of Chinese anesthesiologists toward difficult airways
Source: BMC Med Educ. 2025 May 9;25:683. doi: 10.1186/s12909-025-07264-x (PMC12065192; doi:10.1186/s12909-025-07264-x)
Supplement: Supplementary file 2 — Supplementary Material 2 [file 12909_2025_7264_MOESM2_ESM.docx]

Table S1. Knowledge dimension of the participants.

|  | **Accuracy, n (%)** |
| --- | --- |
| 1. Airway risk assessment and airway examination before anesthesia or airway management are mainly based on physical examination and additional special assessment methods. | 30 (3.02) |
| 1. The Mallampati score can be used to assess the degree of oropharyngeal opening and can be used alone to predict difficult airways. | 477 (48.08) |
| 1. The modified Mallampati score assesses the soft palate, uvula, and fauces as Grade II. | 575 (57.96) |
| 1. The primary method of airway assessment is special assessment methods such as ultrasound and endoscopy. | 385 (38.81) |
| 1. Before implementing general anesthesia, anesthesiologists should ensure that difficult airway tools are prepared in the operating room. | 937 (94.46) |
| 1. Informing patients or their families in advance of the risks and procedures of difficult airway management is part of preparing for difficult airways. | 968 (97.58) |
| 1. Should patients with suspected difficult airways identified during airway assessment be directly managed as difficult airways? | 884 (89.11) |
| 1. The video laryngoscope is currently the most widely used and popular difficult airway intubation tool, but whether it should be the first choice is not yet determined. | 609 (61.39) |
| 1. Awake tracheal intubation is recognized as the gold standard for managing anticipated difficult airways. | 838 (84.48) |
| 1. Awake fiberoptic intubation is most suitable for obese patients with difficult mask ventilation. | 817 (82.36) |
| 1. When encountering difficulties during tracheal intubation, attempting intubation is more important than ensuring ventilation. | 799 (80.54) |
| 1. Actively seeking opportunities for oxygenation while managing difficult airways is important, and oxygenation is unnecessary during extubation. | 913 (92.04) |
| 1. Excessive attempts at tracheal intubation or supraglottic device placement may cause potential damage and complications. The latest ASA guidelines recommend a maximum number of attempts to try different tools and techniques. | 229 (23.08) |
