# Supplementary material for: Knowledge, attitudes, and practices of Chinese anesthesiologists toward difficult airways
Source: BMC Med Educ. 2025 May 9;25:683. doi: 10.1186/s12909-025-07264-x (PMC12065192; doi:10.1186/s12909-025-07264-x)
Supplement: Supplementary file 3 — Supplementary Material 3 [file 12909_2025_7264_MOESM3_ESM.docx]

Table S2. Attitude dimension of the participants.

|  | **N (%)** | | | | |
| --- | --- | --- | --- | --- | --- |
|  | **Strongly agree** | **Agree** | **Neutral** | **Disagree** | **Strongly disagree** |
| 1. You believe assessing the risk of a difficult airway and aspiration before anesthesia or airway management is crucial. P | 895 (90.22) | 85 (8.57) | 11 (1.11) | / | 1 (0.1) |
| 1. You believe that regardless of whether the assessment indicates a difficult airway, it is important to confirm the availability of difficult airway tools before anesthesia. P | 777 (78.33) | 162 (16.33) | 40 (4.03) | 11 (1.11) | 2 (0.2) |
| 1. You strongly resist participating in the anesthesia and management of difficult airway patients due to concerns about causing injury from improper handling. N | 74 (7.46) | 53 (5.34) | 95 (9.58) | 383 (38.61) | 387 (39.01) |
| 1. Although anesthesia or airway management of difficult airways presents certain challenges, you maintain a positive attitude towards managing these patients. P | 733 (73.89) | 233 (23.49) | 25 (2.52) | / | 1 (0.1) |
| 1. You agree that timely summarization and reporting, whether the outcome is positive or challenging, are opportunities for continuous improvement in difficult airway management. P | 828 (83.47) | 148 (14.92) | 13 (1.31) | 2 (0.2) | 1 (0.1) |
| 1. You believe you can handle any situation involving difficult airways and, therefore, do not need assistance. N | 43 (4.33) | 21 (2.12) | 49 (4.94) | 328 (33.06) | 551 (55.54) |
| 1. You believe that human factors are particularly important for difficult airway management personnel, especially in emergencies, and should be given increased attention and learning. P | 823 (82.96) | 148 (14.92) | 14 (1.41) | 5 (0.5) | 2 (0.2) |

P represents positive questions, N represents negative questions, with reverse scoring.
