# Supplementary material for: Knowledge, attitudes, and practices of Chinese anesthesiologists toward difficult airways
Source: BMC Med Educ. 2025 May 9;25:683. doi: 10.1186/s12909-025-07264-x (PMC12065192; doi:10.1186/s12909-025-07264-x)
Supplement: Supplementary file 4 — Supplementary Material 4 [file 12909_2025_7264_MOESM4_ESM.docx]

Table S3. Practice dimension of the participants.

|  | **N (%)** | | | | |
| --- | --- | --- | --- | --- | --- |
|  | **Always** | **Often** | **Sometimes** | **Occasionally** | **Never** |
| 1. Before administering anesthesia or airway management, you assess the risk of difficult airway and aspiration for all patients. P | 767 (77.32) | 197 (19.86) | 24 (2.42) | 1 (0.1) | 3 (0.3) |
| 1. Before administering anesthesia or airway management, you perform airway-related examinations for all patients. P | 603 (60.79) | 248 (25) | 111 (11.19) | 29 (2.92) | 1 (0.1) |
| 1. Before anesthesia or airway management, you check and inquire about the medical records of previous surgical anesthesia experiences related to difficult airways. P | 643 (64.82) | 233 (23.49) | 87 (8.77) | 27 (2.72) | 2 (0.2) |
| 1. You successfully predict and assess the frequency of difficult airways. P | 159 (16.03) | 540 (54.44) | 239 (24.09) | 50 (5.04) | 4 (0.4) |
| 1. If a patient is assessed as having a difficult airway, you check and confirm that the operating room is equipped with difficult airway equipment before administering anesthesia. P | 839 (84.58) | 111 (11.19) | 38 (3.83) | 4 (0.4) | / |
| 1. If a patient is assessed as having a difficult airway, you inform the patient or family in advance of the risks and procedures of difficult airway management. P | 835 (84.17) | 119 (12) | 32 (3.23) | 6 (0.6) | / |
| 1. If a patient is not assessed as having a difficult airway, you may not confirm whether the operating room is equipped with difficult airway equipment before administering anesthesia. N | 122 (12.3) | 177 (17.84) | 210 (21.17) | 231 (23.29) | 252 (25.4) |
| 1. Do you prepare difficult airway equipment and start rapid induction anesthesia for patients assessed as having suspicious difficult airways? P | 361 (36.39) | 180 (18.15) | 199 (20.06) | 148 (14.92) | 104 (10.48) |
| 1. In difficult airways, you monitor the patient’s oxygenation and ventilation status and adjust management strategies promptly. P | 782 (78.83) | 171 (17.24) | 37 (3.73) | 2 (0.2) | / |
| 1. You can make decisions quickly when handling difficult airways. P | 435 (43.85) | 448 (45.16) | 103 (10.38) | 5 (0.5) | 1 (0.1) |
| 1. When encountering unexpected difficult airways, you quickly determine further airway management strategies while ensuring patient oxygenation. P | 539 (54.33) | 352 (35.48) | 92 (9.27) | 8 (0.81) | 1 (0.1) |
| 1. When handling difficult airways, you successfully use awake fiberoptic intubation. P | 129 (13) | 287 (28.93) | 289 (29.13) | 233 (23.49) | 54 (5.44) |
| 1. Even if you can independently manage difficult airways, you still ensure that at least one assistant can assist at any time. P | 762 (76.81) | 188 (18.95) | 36 (3.63) | 3 (0.3) | 3 (0.3) |
| 1. When encountering difficult airways beyond your capability, you actively seek help and advice from colleagues. P | 813 (81.96) | 144 (14.52) | 31 (3.13) | 3 (0.3) | 1 (0.1) |
| 1. You successfully manage difficult airways at a certain frequency. P | 181 (18.25) | 422 (42.54) | 256 (25.81) | 128 (12.9) | 5 (0.5) |

P represents positive questions, N represents negative questions, with reverse scoring.
