# Supplementary material for: Knowledge, attitudes, and practices of Chinese anesthesiologists toward difficult airways
Source: BMC Med Educ. 2025 May 9;25:683. doi: 10.1186/s12909-025-07264-x (PMC12065192; doi:10.1186/s12909-025-07264-x)
Supplement: Supplementary file 5 — Supplementary Material 5 [file 12909_2025_7264_MOESM5_ESM.docx]

Table S4. Fit indices of the structural equation model (SEM) before and after model adjustment.

| **Indicators** | **Ref.** | **Measured results (pre-adjustment)** | **Measured results (model graph after MI index adjustment)** | **Measured results (model graph after baseline adjustment added)** |
| --- | --- | --- | --- | --- |
| CMIN/DF | 1-3 excellent，3-5 good | 5.814 | 4.307 | 4.053 |
| RMSEA | <0.08 good | 0.070 | 0.058 | 0.056 |
| IFI | >0.8 good | 0.624 | 0.745 | 0.730 |
| TLI | >0.8 good | 0.596 | 0.722 | 0.707 |
| CFI | >0.8 good | 0.622 | 0.744 | 0.728 |
