# Supplementary material for: Knowledge, attitudes, and practices of Chinese anesthesiologists toward difficult airways
Source: BMC Med Educ. 2025 May 9;25:683. doi: 10.1186/s12909-025-07264-x (PMC12065192; doi:10.1186/s12909-025-07264-x)
Supplement: Supplementary file 6 — Supplementary Material 6 [file 12909_2025_7264_MOESM6_ESM.docx]

Table S5. Model path parameters after adding baseline information

| **Path** | | | **Estimate** | **S.E.** | **C.R.** | **P** |
| --- | --- | --- | --- | --- | --- | --- |
| Attitude | <--- | Knowledge | 1.360 | 0.792 | 1.717 | 0.086 |
| Attitude | <--- | Successfully_rescued_cases | 0.074 | 0.027 | 2.717 | 0.007 |
| Practice | <--- | Attitude | 0.783 | 0.072 | 10.883 | <0.001 |
| Practice | <--- | Knowledge | 0.221 | 0.254 | 0.871 | 0.383 |
| Practice | <--- | Years_of_experience_in_anesthesia | 0.026 | 0.005 | 4.932 | <0.001 |
| Practice | <--- | Participation_training | 0.089 | 0.019 | 4.614 | <0.001 |
| Practice | <--- | Successfully_rescued_cases | 0.092 | 0.033 | 2.776 | 0.006 |
| K13 | <--- | Knowledge | 1.000 |  |  |  |
| K12 | <--- | Knowledge | 5.849 | 3.337 | 1.753 | 0.080 |
| K11 | <--- | Knowledge | 8.707 | 4.968 | 1.753 | 0.080 |
| K10 | <--- | Knowledge | 0.487 | 0.457 | 1.064 | 0.287 |
| K9 | <--- | Knowledge | 0.079 | 0.370 | 0.215 | 0.830 |
| K8 | <--- | Knowledge | -3.197 | 1.908 | -1.676 | 0.094 |
| K7 | <--- | Knowledge | 0.006 | 0.354 | 0.016 | 0.987 |
| K6 | <--- | Knowledge | 0.588 | 0.368 | 1.598 | 0.110 |
| K5 | <--- | Knowledge | 0.194 | 0.277 | 0.701 | 0.483 |
| K4 | <--- | Knowledge | 4.255 | 2.491 | 1.708 | 0.088 |
| K3 | <--- | Knowledge | -4.385 | 2.565 | -1.710 | 0.087 |
| K2 | <--- | Knowledge | 4.628 | 2.694 | 1.718 | 0.086 |
| K1 | <--- | Knowledge | -1.631 | 0.967 | -1.687 | 0.092 |
| P1 | <--- | Practice | 1.000 |  |  |  |
| P2 | <--- | Practice | 1.045 | 0.103 | 10.186 | <0.001 |
| P3 | <--- | Practice | 1.181 | 0.092 | 12.907 | <0.001 |
| P4 | <--- | Practice | 0.921 | 0.097 | 9.505 | <0.001 |
| P5 | <--- | Practice | 0.979 | 0.071 | 13.757 | <0.001 |
| P6 | <--- | Practice | 0.912 | 0.070 | 13.033 | <0.001 |
| P7 | <--- | Practice | 0.732 | 0.159 | 4.613 | <0.001 |
| P8 | <--- | Practice | 0.090 | 0.163 | .552 | 0.581 |
| P9 | <--- | Practice | 1.157 | 0.077 | 15.118 | <0.001 |
| P10 | <--- | Practice | 1.227 | 0.093 | 13.168 | <0.001 |
| P11 | <--- | Practice | 1.220 | 0.095 | 12.883 | <0.001 |
| P12 | <--- | Practice | 1.041 | 0.134 | 7.751 | <0.001 |
| P13 | <--- | Practice | 0.993 | 0.077 | 12.829 | <0.001 |
| P14 | <--- | Practice | 0.827 | 0.068 | 12.127 | <0.001 |
| P15 | <--- | Practice | 0.919 | 0.113 | 8.100 | <0.001 |
| A1 | <--- | Attitude | 1.000 |  |  |  |
| A2 | <--- | Attitude | 1.063 | 0.100 | 10.628 | <0.001 |
| A3 | <--- | Attitude | 1.518 | 0.187 | 8.139 | <0.001 |
| A4 | <--- | Attitude | 1.583 | 0.094 | 16.774 | <0.001 |
| A5 | <--- | Attitude | 1.548 | 0.086 | 17.923 | <0.001 |
| A6 | <--- | Attitude | 1.243 | 0.157 | 7.936 | <0.001 |
| A7 | <--- | Attitude | 1.250 | 0.085 | 14.634 | <0.001 |
