# Supplementary material for: Knowledge, attitudes, and practices of Chinese anesthesiologists toward difficult airways
Source: BMC Med Educ. 2025 May 9;25:683. doi: 10.1186/s12909-025-07264-x (PMC12065192; doi:10.1186/s12909-025-07264-x)
Supplement: Supplementary file 7 — Supplementary Material 7 [file 12909_2025_7264_MOESM7_ESM.doc]

Questionnaire No.

| Dear friends:  We are researchers from ** Hospital. We sincerely invite you to participate in our research project. This study aims to understand the **knowledge, attitudes, and practices of Chinese anesthesiologists toward difficult airways**, to serve as the basis for developing scientific intervention strategies, which may help many others in the future to improve their health conditions. Your participation in this study is voluntary, and the research has been approved by the Ethics Review Committee. If you agree to participate, please read the following instructions:  1. Please complete the questionnaire. There are no right or wrong answers; you only need to provide responses based on your actual experiences. If you have any questions during the process, feel free to reach out to us, and please submit the completed questionnaire in a timely manner.  2. This study is a simple questionnaire survey and will not cause any harm to your physical or psychological well-being. However, it may involve some personal information such as your gender and age. Please rest assured that we will strictly maintain confidentiality and will not disclose your information.  3. As a participant, you can always stay informed about the information and progress related to this study. If you decide to withdraw from the study, please let us know, and your data will not be included in the research results.  Finally, we sincerely thank you for taking the time to support our scientific research amid your busy schedule!  £I have been informed and agreed to the use of the collected data for scientific research.  Informed Consent Signature：  Date of participation：YYYY MM DD |
| --- |

| **Part I Basic Information** | | | | |
| --- | --- | --- | --- | --- |
| 1. **Your age:** | **years** | | | |
| 1. **Your gender：** | | A. Male | B. Female |  |
| 1. **Your education：** | | A. Associate degree  B. Bachelor’s degree  C. Master’s degree and above | | |
| **4. Your title:** | | A. No title  B. Junior  C. Intermediate  D. Associate senior  E. Senior | | |
| **5. Nature of your institution:** | | A. Public Level One  B. Public Level Two  C. Public Level Three  D. Private Hospital | | |
| **6. Your years of experience in anesthesia:** | | years | | |
| **7. In the last six months, have you participated in training on difficult airway assessment and management** | | A. Yes  B. No | | |
| **8. In your practice experience, have you encountered cases where patient death was caused by a difficult airway?** | | A. Yes  B. No | | |
| **10. In your practice experience, have you encountered cases where surgery was stopped due to a difficult airway?** | | A. Yes  B. No | | |
| **11. In your practice experience, have you encountered cases of difficult airways and successfully rescued?** | | A. Yes  B. No | | |
| **12. The frequency of encountering difficult airway patients in your department:** | | A. Six-monthly or longer  B. Quarterly  C. Monthly  D. Weekly  E. Daily | | |
| **13. In the last 6 months, has your department encountered cases where patient death was caused by a difficult airway?** | | A. Yes  B. No | | |
| **14. In the last 6 months, has your department encountered cases where surgery was stopped due to a difficult airway?** | | A. Yes  B. No | | |

| **Part II Knowledge of difficult airways** | | | |
| --- | --- | --- | --- |
| **Please judge the following descriptions according to your current knowledge, if you are unable to judge you can choose the unclear option** | | | |
| 1. **Airway risk assessment and airway examination before anesthesia or airway management are mainly based on physical examination and additional special assessment methods.** | A.Yes | B.No | C.Unsure |
| 1. **The Mallampati score can be used to assess the degree of oropharyngeal opening and can be used alone to predict difficult airways.** | A.Yes | B.No | C.Unsure |
| 1. **The modified Mallampati score assesses the soft palate, uvula, and fauces as Grade II.** | A.Yes | B.No | C.Unsure |
| 1. **The primary method of airway assessment is special assessment methods such as ultrasound and endoscopy.** | A.Yes | B.No | C.Unsure |
| 1. **Before implementing general anesthesia, anesthesiologists should ensure that difficult airway tools are prepared in the operating room.** | A.Yes | B.No | C.Unsure |
| 1. **Informing patients or their families in advance of the risks and procedures of difficult airway management is part of preparing for difficult airways.** | A.Yes | B.No | C.Unsure |
| 1. **Should patients with suspected difficult airways identified during airway assessment be directly managed as difficult airways?** | A.Yes | B.No | C.Unsure |
| 1. **The video laryngoscope is currently the most widely used and popular difficult airway intubation tool, but whether it should be the first choice is not yet determined.** | A.Yes | B.No | C.Unsure |
| 1. **Awake tracheal intubation is recognized as the gold standard for managing anticipated difficult airways.** | A.Yes | B.No | C.Unsure |
| 1. **Awake fiberoptic intubation is most suitable for obese patients with difficult mask ventilation.** | A.Yes | B.No | C.Unsure |
| 1. **When encountering difficulties during tracheal intubation, attempting intubation is more important than ensuring ventilation.** | A.Yes | B.No | C.Unsure |
| 1. **Actively seeking opportunities for oxygenation while managing difficult airways is important, and oxygenation is unnecessary during extubation.** | A.Yes | B.No | C.Unsure |
| 1. **Excessive attempts at tracheal intubation or supraglottic device placement may cause potential damage and complications. The latest ASA guidelines recommend a maximum number of attempts to try different tools and techniques.** | A.3 | B.3+1 | C.Unsure |

| **Part III Attitude to difficult airways** | |
| --- | --- |
| 1. **You believe assessing the risk of a difficult airway and aspiration before anesthesia or airway management is crucial.** | A.Strongly agree  B.Agree  C.Neutral  D.Disagree  E.Strongly disagree |
| 1. **You believe that regardless of whether the assessment indicates a difficult airway, it is important to confirm the availability of difficult airway tools before anesthesia.** | A.Strongly agree  B.Agree  C.Neutral  D.Disagree  E.Strongly disagree |
| 1. **You strongly resist participating in the anesthesia and management of difficult airway patients due to concerns about causing injury from improper handling.** | A.Strongly agree  B.Agree  C.Neutral  D.Disagree  E.Strongly disagree |
| 1. **Although anesthesia or airway management of difficult airways presents certain challenges, you maintain a positive attitude towards managing these patients.** | A.Strongly agree  B.Agree  C.Neutral  D.Disagree  E.Strongly disagree |
| 1. **You agree that timely summarization and reporting, whether the outcome is positive or challenging, are opportunities for continuous improvement in difficult airway management.** | A.Strongly agree  B.Agree  C.Neutral  D.Disagree  E.Strongly disagree |
| 1. **You believe you can handle any situation involving difficult airways and, therefore, do not need assistance.** | A.Strongly agree  B.Agree  C.Neutral  D.Disagree  E.Strongly disagree |
| 1. **You believe that human factors are particularly important for difficult airway management personnel, especially in emergencies, and should be given increased attention and learning.** | A.Strongly agree  B.Agree  C.Neutral  D.Disagree  E.Strongly disagree |

| **Part IV Practice on difficult airways** | | | | | |
| --- | --- | --- | --- | --- | --- |
| 1. **Before administering anesthesia or airway management, you assess the risk of difficult airway and aspiration for all patients.** | A.Always | B.Often | C.Sometimes | D.Occasionally | E.Never |
| 1. **Before administering anesthesia or airway management, you perform airway-related examinations for all patients.** | A.Always | B.Often | C.Sometimes | D.Occasionally | E.Never |
| 1. **Before anesthesia or airway management, you check and inquire about the medical records of previous surgical anesthesia experiences related to difficult airways.** | A.Always | B.Often | C.Sometimes | D.Occasionally | E.Never |
| 1. **You successfully predict and assess the frequency of difficult airways.** | A.Always | B.Often | C.Sometimes | D.Occasionally | E.Never |
| 1. **If a patient is assessed as having a difficult airway, you check and confirm that the operating room is equipped with difficult airway equipment before administering anesthesia.** | A.Always | B.Often | C.Sometimes | D.Occasionally | E.Never |
| 1. **If a patient is assessed as having a difficult airway, you inform the patient or family in advance of the risks and procedures of difficult airway management.** | A.Always | B.Often | C.Sometimes | D.Occasionally | E.Never |
| 1. **If a patient is not assessed as having a difficult airway, you may not confirm whether the operating room is equipped with difficult airway equipment before administering anesthesia.** | A.Always | B.Often | C.Sometimes | D.Occasionally | E.Never |
| 1. **Do you prepare difficult airway equipment and start rapid induction anesthesia for patients assessed as having suspicious difficult airways?** | A.Always | B.Often | C.Sometimes | D.Occasionally | E.Never |
| 1. **In difficult airways, you monitor the patient’s oxygenation and ventilation status and adjust management strategies promptly.** | A.Always | B.Often | C.Sometimes | D.Occasionally | E.Never |
| 1. **You can make decisions quickly when handling difficult airways.** | A.Always | B.Often | C.Sometimes | D.Occasionally | E.Never |
| 1. **When encountering unexpected difficult airways, you quickly determine further airway management strategies while ensuring patient oxygenation.** | A.Always | B.Often | C.Sometimes | D.Occasionally | E.Never |
| 1. **When handling difficult airways, you successfully use awake fiberoptic intubation.** | A.Always | B.Often | C.Sometimes | D.Occasionally | E.Never |
| 1. **Even if you can independently manage difficult airways, you still ensure that at least one assistant can assist at any time.** | A.Always | B.Often | C.Sometimes | D.Occasionally | E.Never |
| 1. **When encountering difficult airways beyond your capability, you actively seek help and advice from colleagues.** | A.Always | B.Often | C.Sometimes | D.Occasionally | E.Never |
| 1. **You successfully manage difficult airways at a certain frequency.** | A.Always | B.Often | C.Sometimes | D.Occasionally | E.Never |
